# Supplementary material for: Are carbon emissions trading and green financial instruments synergistic? -Comprehensive quantitative research based on content analysis
Source: PLoS One. 2024 Mar 7;19(3):e0298601. doi: 10.1371/journal.pone.0298601 (PMC10919601; doi:10.1371/journal.pone.0298601)
Supplement: S1 Data — (ZIP) [file pone.0298601.s001.zip › Data/地级市Z_F工作报告.zip!/╣π╢1⁄2/╒┐╜¡╩╨/└┤╫╘╣1⁄2╓┌║┼í╛┬φ┐╦╩2╛▌═°í┐_2011.docx]

各位代表：

　　我代表市人民政府向大会作政府工作报告，请予审议，并请市政协委员和其他列席的同志提出意见。

　　2010年及“十一五”时期工作回顾

　　2010年是全面完成“十一五”规划的冲刺之年。一年来，在中共湛江市委的正确领导下，在市人大及其常委会的监督和市政协的支持下，市政府以科学发展观为统领，紧紧围绕争当粤西地区振兴发展龙头的目标，深入实施“工业立市、港口兴市、生态建市”发展战略，抢抓机遇，发挥优势，科学务实，攻坚克难，顺利完成了市十二届人大六次会议和“十一五”发展规划确定的各项目标任务，经济社会发展取得了显著成就。全市生产总值1402.77亿元，增长14.2%，创15年来最高增速，人均生产总值20048元，增长13.4%；三次产业结构调整为21∶41.1∶37.9；地方财政一般预算收入66.23亿元，增长25.79%；来源于湛江的财政总收入341.14亿元，增长35.67%。

　　——这是“三次产业”进一步优化的一年

　　工业化进程加快。规模以上工业总产值1405.97亿元,增长25.1%;增加值464.06亿元,增长18.2%；海洋产业总产值713.8亿元、增加值336.8亿元，分别增长10%和8%；规模以上工业经济效益综合指数370.1%，提高77.5个百分点。14个项目入选全省现代产业500强，总投资1382亿元，居全省第二位。广钢环保迁建湛江和中科炼化一体化项目前期工作加快推进；晨鸣林浆纸一体化项目超额完成年度投资计划，将于今年上半年建成投产；奥里油电厂油改煤、广东粤电湛江生物质发电、国电雷州东里风电场等5个项目动工建设。产业转移工作在省考核中获得优秀成绩，累计承接产业转移项目259项，总投资2505.64亿元，已有130多个项目建成投产。廉江家电和吴川羽绒产业集群被认定为省级产业集群升级示范区。组团参加省第六届“山洽会”，签约项目金额29亿元。

　　服务业显著提升。服务业增加值531.65亿元，增长15.2%。港口吞吐量1.36亿吨，增长15.2%；集装箱吞吐量32.02万标箱，增长38.2%。铁路货物发运量2790.66万吨，增长9%；民航旅客吞吐量48.43万人次，增长22%。建成市、县两级各类专业服务机构400多家，其中9家被认定为省级示范机构。地方金融业发展提速，光大银行、交通银行进入湛江；市商业银行重庆、长沙分行开业，成为我省首家跨省经营的城市商业银行，被评为“全国最具竞争力中小银行”；6家小额贷款公司获省批准运营，新增担保总额23.7亿元，居全省前列；全市金融机构人民币存款余额1556亿元、贷款余额714.4亿元，分别比年初增长19.1%、16.5%。保险业服务领域不断拓宽，保险费收入30.35亿元，增长30.6%，政策性水稻种植保险面积居全省第一，政策性农村住房保险覆盖率达到100%。旅游业在建超亿元项目25个，完成投资36.2亿元，增长182%，居全省首位；粤西明珠美食广场、吴川吉兆湾国际海洋度假中心等16个项目建成；特呈岛被命名为省首批红色旅游基地，东海岛旅游区、雷州天成台、吴川吉兆湾被评为省滨海旅游示范区，徐闻大汉三墩被列为省森林生态旅游示范基地；成功举办第四届海上国际龙舟邀请赛、第三十七届世界旅游小姐全球总决赛；全年接待游客1415万人次、旅游业总收入65.3亿元，分别增长23.8%和22.3%。圆满举办省首届海洋经济博览会，我市签约项目金额102.5亿元。荣获“中国对虾之都”称号。连续7年被评为中国商业百强城市。

　　现代农业稳步发展。农业总产值471.42亿元，增长6.9%，居全省前列。粮食、蔬菜、糖蔗、香蕉、菠萝、花卉等主要农作物及水产品产量均为全省之冠。订单农业发展迅速，签订合同金额85.55亿元，连续4年居全省第一；新增省级和市级农业龙头企业各2家、农民专业合作社439家；湛江国联水产成为我市首家上市农业龙头企业。新建省级现代农业示范区1个、现代农业园区3个。总投资逾80亿元的正大（湛江）农业综合开发基地项目顺利推进。农业标准化水平居全省前列，新增1个国家级、4个省级农业标准化示范区，新建现代标准农田11万亩和省级标准化生猪养殖场10个，建成国家级产业技术试验站，制定36项省、市级农业地方标准、300多项农产品标准及350多项农业生产资料标准，初步建立起与国家、行业、省地方标准相互配套的农业标准体系。新增省级农业名牌产品12个、绿色食品认证14个、有机食品认证2个。开发补充耕地17.8万亩，名列全省第一；连续11年实现耕地占补平衡，受到省表扬。农村土地流转157.99万亩，占农用土地总面积22%。农机拥有量居全省前列。全国首个国家级珍珠种苗良种场获得批准，全省首个深水网箱养殖产业园区落户；硇洲、乌石国家中心渔港主体工程基本完成，龙头沙国家一级渔港项目获农业部立项。31宗省城乡水利防灾减灾工程完成投资19.15亿元，完成率58.5%；118宗列入省病险水库加固工程完成投资2.89亿元，完成率70.1%。

　　——这是“三大需求”进一步扩大的一年

　　投资拉动强劲。完成重点项目投资209.1亿元，增长65.6%。在重点项目建设有力带动下，完成全社会固定资产投资526.57亿元，增长33.9%。湛江港30万吨级液体化工码头、成品油码头改造和15万吨级散货码头升级改造等工程竣工；湛徐高速公路、东海岛跨海大桥、国道325线文车至北罗坑段一级公路建成通车；茂湛铁路、海湾大桥连接线二期工程等动工建设；雷州青年运河灌区续建配套与节水改造和鉴江供水枢纽工程加快推进。

　　消费持续畅旺。社会消费品零售总额679.79亿元，增长21.8%。城乡消费服务体系不断完善，建成大型商品零售网点25个，培育年成交额超亿元以上批发市场21家；建设县级配送中心13个、镇（村）级农家店2192个，覆盖率分别达100%和84%。全年住房公积金用于购买和改善住房19.66亿元，有力促进住房消费。汽车、教育、文化、运动健身等新型消费形成热点。农村消费扩大，农机、家电等下乡实现销售额3.67亿元，增长1.5倍。

　　外经贸恢复性增长。外贸进出口总额35.32亿美元，增长25.3%；其中出口16.87亿美元，增长23.6%，进口18.45亿美元，增长27%。43家水产品加工企业获得欧盟、美国等出口卫生认证，列全省第一。新批外商投资项目13个，增长62.5%；合同外资1.05亿美元，增长11.5%；实际吸收外资3671万美元，增长25.5%。

　　——这是“三个支撑”进一步增强的一年

　　体制改革支撑增强。完成新一轮政府系统机构改革和行政事业单位分类改革。开展第四轮行政审批制度改革，审批事项精简50%以上、三分之一下放到县（市），促进政府职能转变。不失时机推进国有企业改革创新，湛江港集团继引入香港招商局集团、上海宝钢集团成功改制后，又与中石化、中石油等央企建立战略合作伙伴关系；徐闻港区海安港与海安新港实现联营；引入中纺粮油集团，收购盘活富虹、华农两大民营食用油加工企业；冠豪纸业引入中物投资公司投资40亿元加盟控股经营，成为股权引资的成功范例。实施投资体制改革，组建市城市基础设施建设投资集团、市交通投资集团和市水务集团等投融资平台，有力支持了交通和城市建设。成功收购湛江海湾大桥股权。推进医疗药品价格改革试点和污水、垃圾处理收费改革。制定实施市对经济技术开发区财政管理调整政策，在全省率先开展市直事业单位财务预算管理。“广东（湛江）统筹城乡发展金融改革创新综合试验区”建设步伐加快，分别被评为“中国最具特色金融生态示范城市”和“广东省金融稳定市”；稳妥推进农村信用社第二轮改革。集体林权主体改革基本完成，已确权发证97.9%。创新城乡居民基本医疗保险一体化体制机制，综合运用行政和现代金融保险资源，探索出受到国务院和省委、省政府领导充分肯定的湛江医保新路子。民营经济增加值678.37亿元，增长18.7%，占生产总值比重达到48.4%，成为拉动经济增长的重要力量。

　　自主创新支撑增强。新增国家高新技术企业12家；认定市级自主创新企业16家、自主创新培育企业19家。实施高新技术项目107项，28个高新技术产品认定为省级自主创新产品。获批设立省级高新技术产业开发区。新增4家省级企业技术中心、1家省级工程中心和2家省级农业创新中心；建立各类企业技术开发机构80多家，新增产学研合作项目33项。专利授权量增长66%；获得科技成果51项，其中22项转化为企业标准。注册商标、省著名商标、中国驰名商标均居粤西首位。高新技术产品总产值220亿元，增长10%。

　　生态环境支撑增强。单位生产总值能耗超额完成年度指标任务。全市建成污水处理厂7座，日处理能力33万吨，县（市）城区污水处理厂全部竣工投产，市区污水处理率逾80%。主要污染物二氧化硫、化学需氧量两项减排提前实现省下达的指标。创建国家环保模范城市考核指标达标19项，占规定考核指标的73%。空气环境质量继续名列全国环境保护重点城市前列。饮用水源和近岸海域水质达标率继续保持100%。造林20.6万亩，完成任务103%；森林覆盖率28.3%；徐闻成功创建“广东省林业生态县”；新增2个省级生态示范镇，200条自然村建成“林业生态文明村”。

　　——这是“三项建设”进一步加快的一年

　　城市建设加快。编制完成《土地利用总体规划（2010-2020年）》、《城市综合交通规划》、《市区“三旧”改造专项规划》、《市区绿道网规划》以及市区环境与卫生、城市照明等专项规划；编制实施东海岛、麻章区和南三岛等一批控制性详细规划。数字地理空间框架建设被国家批准为试点市。扩大土地储备，收储土地6600亩。完成人民大道改造和海田路景观整治二期、赤坎江整治二期等工程；动工建设滨湖路二期工程、新湖大道和奋勇大道；稳步推进湛江大道、银帆公园、麻章公园等项目前期工作；人民大道改造工程荣获国家市政工程“金杯奖”。更新462辆天然气双燃料出租汽车，新增190辆液化天然气小型公交车和公交大巴，市民出行和城市交通条件明显改善。实施“三旧”改造项目109个，投入资金49.1亿元，霞山渔人码头动工建设。实施三岭山森林公园生态恢复工程，扩大面积1.18万亩。新增城市绿地103万平方米。绿塘河湿地公园被命名为“国家城市湿地公园”。荣获“中国城乡建设范例城市”、“中国十佳绿色城市”、“中国十大绿色生态城市”和“全国人民防空先进城市”等称号。

　　民生社会建设加快。全年用于社会民生事业的财政资金107.24亿元，比上年增加24.63亿元，增长29.82%，占地方财政一般预算支出的69.8%，是社会民生投入力度最大、城乡居民受益最多的一年。城镇居民人均可支配收入15305元，增长12%；农民人均纯收入6906元，增长17.2%，增速创12年来新高。年初确定的18件民生实事全面落实。基本完成小街小巷改造和路灯安装；城镇失业人员再就业4.2万人，其中就业困难对象再就业2188人；新增城镇就业6.6万人；开展农村劳动力技能培训5.5万人，新增转移农村劳动力就业16.7万人。养老保险参保66.3万人、失业保险参保34.1万人、城镇职工医疗保险参保49.1万人、工伤保险参保28.53万人、生育保险参保34.03万人；城乡居民基本医疗保险参保587.5万人，提前完成省医改参保目标。全额资助低保户、五保户等困难群众22.4万人参加城乡居民医疗保险。19万企业离退休人员基本养老金按时足额发放。累计支出城乡居民低保补差金1.76亿元，发放市区低保和五保户物价补贴600万元，有效保障困难群众基本生活。全面完成省下达的75.73万人饮水安全工程建设任务。建设廉租房365套、经济适用住房158套。因灾房屋全倒的1930户新房全部建成入住。建成水库移民新村85条，解决4000户、1.8万人住房困难。完成121间村镇敬老院建设。10间乡镇卫生院改造、20户以上已通电自然村广播电视“村村通”、784个农家书屋和17条农村民居地震安全示范村全部完成。解决老区村庄5.6万人行路难和2.8万人饮水难问题，完成2个老区村庄近200人搬迁安置。扶贫开发“双到”工作达到省优秀水平，富有成效开展“广东扶贫济困日”活动，筹集社会扶贫资金1.08亿元，364个单位对355个贫困村投入帮扶资金4.87亿元，改造贫困村民危房2万多户，建成帮扶项目710个，带动8万多贫困人口年人均增收2500元以上。社会事业全面进步。完成第二批1814名“代转公”教师招录和2100多名代课教师转岗、辞退工作；全面实施教师绩效工资；高中阶段毛入学率86.5%，提前一年实现基本普及高中阶段教育；高考本科上线人数突破1.7万人，再创历史新高；启动湛江职教基地建设。开展湛江文化建设定位和湛江人文精神大讨论，组织特色节目参加上海世博会“广东周”及港澳台等对外文化艺术交流演出获广泛好评，群众文化和开心广场活动推广取得成效，成功举办动漫文化节；新增省级文物保护单位7处、市级文物保护单位29处和省级以上非物质文化遗产项目7个；徐闻县雷剧团获得“全国服务农民服务基层文化建设先进集体”殊荣。启动中央扶持的25个卫生项目、15项基本或重大公共卫生服务项目；城市社区和行政村卫生服务站覆盖率分别达到98%和96%。湛江籍运动员何冲在第十六届亚运会勇夺男子跳水3米跳板冠军，刘鸥、陈晓君获花样游泳集体自由自选和组合冠军；2名选手参加第十届亚残运会均夺得奖牌，谭业腾获赛艇男子单人双桨1000米冠军；在第十三届省运会上获金牌总数和团体总分排名全省第八位，荣获“突出贡献奖”、“体育道德风尚奖”；成功举办第十一届“体育节”和第四届海上国际龙舟邀请赛。完成年度人口与计生工作任务，人口出生率11.82‰，自然增长率7.23‰；赤坎区被评为全国计划生育优质服务先进单位。顺利开展第六次全国人口普查。强化价格调控和服务，在全省率先运用价格认证手段服务重点项目建设，为项目节省资金1000多万元。对口支援汶川龙溪乡地震灾区恢复重建54个项目全面完成。出台《加快残疾人事业发展的实施意见》，加大对残疾人援助。成功举行地震和粮食应急演练。双拥、气象、海防、档案、供销社、地方志、社科、民族宗教、妇女儿童、老龄和国家安全等工作取得新成绩。

　　民主法制建设加快。全年办理省市人大代表议案、建议和政协委员提案共349件，办复率均达100%。实现行政审批和重点工作、重点项目全程监督，拟建项目建立管理档案并向社会公告，重点工作按时办结率达到97%，重点项目按时间节点完成率达到95%，提高20个百分点，市直单位审批事项提前办结率100%。规范性文件报备率和及时率、行政执法投诉结案率均达100%。深入开展纠风治乱工作，强化惠农资金监管。严格食品市场准入，查处假冒伪劣产品案件3190宗，有效保障群众生活安全。保持打私高压态势，连续8年没有发生暴力抗拒缉私和群体性走私案件。“五五”普法教育工作走在全省前列，创建省、市民主法治村（社区）226个，徐闻县被评为全国法治县创建活动先进单位。充分发挥审计推进依法理财、促进廉政建设作用，完成审计或审计调查项目166个。政务公开工作在全省考核中获得优秀成绩。开展违法建设专项整治，拆除违法建筑24.39万平方米。坚持市政府领导到军营办公，为驻军部队解决实际困难问题13件；投入4700万元建设国防综合训练基地，投入1200万元加强基层武装部和民兵基础建设。“强综治、创平安、促发展”主题活动扎实推进，县、镇、村三级综治信访维稳中心（站）全部建成并有效运作，群众集体上访大幅下降，霞山区被评为省综治维稳先进单位；扎实开展严打整治，社会治安继续向好；市区交通综合治理行动初见成效；全面落实企业安全生产主体责任，集中整治重大安全隐患，人民群众安全感进一步提升。

　　各位代表：2010年各项目标任务的顺利完成，为湛江“十一五”发展划上了圆满句号。站在五年时间节点上回眸，“十一五”时期是我市历史进程中极不平凡的时期，是全市经济实力提升最快、重大项目建设投资最多、城乡面貌深刻变化、人民生活大幅提高的五年，实现了八个主要经济指标翻番：一是生产总值翻一番。由2005年的681亿元增至2010年的1402.77亿元，比“十五”期末增长1.1倍，相当于五年再造一个湛江经济。二是规模以上工业总产值和利税总额翻一番。分别由2005年的640亿元和104.55亿元增至2010年的1405.97亿元和214.72亿元，均比“十五”期末增长1倍多，相当于五年再造一个湛江工业。三是服务业增加值翻一番。由2005年的222.66亿元增至2010年的531.65亿元，比“十五”期末增长1.4倍。四是全社会固定资产投资翻一番。由“十五”时期累计628亿元增至“十一五”时期累计1665亿元，增长1.65倍。五是港口吞吐量翻一番。由2005的6620万吨增至2010年的1.36亿吨，比“十五”期末增长1.1倍。六是社会消费品零售总额翻一番。由2005年的269.98亿元增至2010年的679.79亿元，比“十五”期末增长1.52倍。七是旅游业总收入和游客流量翻一番。分别由2005年的32亿元和560万人次增至2010年的65.3亿元和1415万人次，比“十五”期末增长1倍和1.5倍。八是财政总收入翻一番。由2005年的117.85亿元增至2010年的341.14亿元，比“十五”期末增长1.9倍。“十一五”时期取得的重大成就，将对我市“十二五”及长远发展产生具有决定性意义的影响：一是总投资近1300亿元的湛江钢铁和中科炼化一体化两大项目落户并被国家批准开展前期工作，实现了改革开放以来全市重大项目建设的历史性突破，为我市发挥后发优势、实现跨越发展提供了广阔空间和强大后劲；二是面对国际金融危机的影响和冲击，我市经济不仅没有发生大的波动，而是逆势上扬，生产总值增速连续三年超越全省平均水平，这一转折性的发展态势，对全市干部群众坚定后发崛起、争当区域发展龙头信心是巨大的鼓舞；三是经过五年来的努力，我市发展思路、发展战略不断完善，基础设施承载力不断增强，加上相当于前10个五年计划投资总和的1665亿元固定资产投资形成和积聚的巨大能量，为厚积薄发、持续快速发展奠下了坚实基石。这一切，将在物质和精神上，成为我们乘胜前进，开创“十二五”又好又快发展新局面的强力支撑。

　　各位代表，这一切成绩来之不易，是中共湛江市委正确领导下全市人民团结奋斗的结果，凝聚着社会各界的智慧和辛劳。在此，我代表市人民政府，向各级人大代表和政协委员，向全市广大工人、农民、知识分子、干部职工，向驻湛人民解放军、武警官兵、人民警察和各民主党派、人民团体、社会各界人士，向长期关心支持我市改革发展的港澳台同胞、海外侨胞、国际友人，表示崇高的敬意和衷心的感谢!

　　在充分肯定成绩、增强发展信心的同时，我们也清醒认识到，我市经济社会发展还存在一些较为突出的矛盾和问题：一是科学发展与产业结构不够合理的矛盾，先进制造业和战略性新兴产业规模不大、服务业层次不高，产业转型升级的任务繁重；二是科学发展与城乡区域不够协调的矛盾，中心城区辐射带动力不强，县域经济发展不平衡，农村集体经济薄弱；三是科学发展与资源、环境容量制约的矛盾，项目用地紧张，环境承载力不适应新一轮大投资大开发需求日益凸显；四是科学发展与科技、人才滞后的矛盾，产业竞争力特别是自主创新能力不强，产业附加值总体上仍处于国内外产业链的低端；五是科学发展与城市建设管理相对落后的矛盾，旧城区和城中村改造有待加快，交通秩序和违法建设问题有待进一步治理；六是科学发展与民生保障能力不强的矛盾，就业、社保、教育、文化、医疗、扶贫、社会治安等方面仍存在不少亟待解决的困难。此外，政府职能和机关作风有待进一步转变，一些部门领导干部推动科学发展的能力不足、办法不多，主动服务意识淡薄，办事效率不高，与新时期、新形势、新任务的要求不相适应，等等。我们必须高度重视这些矛盾和问题，进一步增强忧患意识、竞争意识、责任意识，采取更加有力措施加以解决。

　　“十二五”时期奋斗目标和2011年工作安排

　　“十二五”时期，是我市承担全省区域协调发展重任、争当粤西地区振兴发展龙头、全面建设小康社会的关键时期，是实现科学发展、后发崛起的重要机遇期。根据中共湛江市委九届十二次会议的决定，市政府编制了《湛江市国民经济和社会发展第十二个五年规划纲要》（草案），提交本次大会审议。

　　加快“十二五”时期发展，必须围绕一个目标：基本确立粤西地区振兴发展龙头地位。必须抓住两个关键：一是坚持以科学发展为主题。全面深化对湛江市情及发展阶段、发展定位、发展规律的认识和把握，始终坚持发展第一要务不动摇，按照科学发展的要求，努力实现有质量、有效益、可持续的快速发展，千方百计扩大经济总量，以总量的扩张带动调结构促转变，夯实建设幸福湛江的物质基础。二是坚持以“转变发展方式、建设幸福湛江”为核心。坚定不移贯彻落实科学发展观，牢牢把握“十二五”发展主线，通过加快转变发展方式增强湛江经济社会发展的均衡性、协调性、可持续性和核心竞争力，不断创造和公平分配社会财富，让全市人民共享发展成果，过上更加幸福生活。必须坚持“五个突出”：一是突出抓好现代产业体系建设。以培育“现代产业50强”和湛江钢铁、中科炼化一体化等龙头型项目建设为重点，努力做强做大以钢铁、石化为引领的先进制造业，以港口物流和特色旅游为引领的现代服务业，以农海产品精深加工为引领的现代农业，以临港工业为引领的现代海洋产业，建设海洋经济强市，加快构建结构优化、附加值高、竞争力强的现代产业体系。二是突出抓好扩大有效需求。坚持求发展与调结构相结合、扩内需与稳外需相结合、增投资与促消费相结合，举全市之力推进总投资4700亿元的基础设施、现代产业、宜居城乡、绿色生态、民生保障、文化强市六大工程项目建设，着力培育和扩大消费需求，提升对外贸易，不断擦亮国家沿海开放城市品牌，加快形成消费、投资、出口协调拉动经济增长新局面。三是突出抓好统筹城乡区域发展。坚持联动推进县域工业化、农业产业化、新型城镇化和新农村建设，加快发展和壮大县域经济，着力建设宜居城乡，不断增强以工促农、以城带乡能力，加速城乡一体化进程。四是突出抓好改革创新。推进重点领域与关键环节体制改革，着力构建有利于争当区域发展龙头的体制机制；深入实施科教人才强市战略，加快建设创新型城市，实现经济增长从要素驱动向创新驱动转变。五是突出抓好以改善民生为重点的社会建设。完善保障和改善民生的制度安排，加快发展社会事业，完善公共服务体系，努力保障人民权益，优化人居环境，促进社会公平正义，维护社会和谐稳定，不断增强人民群众的幸福感。

　　“十二五”时期我市经济社会发展主要目标：综合实力明显提升。主要经济指标增速高于全国、全省平均水平，争当粤西地区振兴发展龙头“三年大变化、十年大跨越”的阶段性目标取得决定性进展。全市生产总值年均增长13%，人均生产总值年均增长11.3%；地方财政一般预算收入年均增长13.3%。结构调整成效明显提升。固定资产投资年均增长18%；社会消费品零售总额年均增长16%；期末三次产业比例优化为15∶47∶38。基本建成以钢铁、石化、造纸为龙头的现代产业体系，内需主导、消费驱动的增长模式初步形成，先进制造业、高新技术产业和现代服务业比重大幅提升；海洋经济成为新增长点，港口货物吞吐量超2.5亿吨；开放型经济达到新水平，外贸进出口年均增长10%，累计实际利用外资6.3亿美元以上。改革创新活力明显提升。国有企业、财政金融、要素价格等重点领域和关键环节改革取得明显进展，政府职能加快转变，创业创新形成新局面，非公有制经济发展提速，占生产总值比重超过50%。文化软实力明显提升。城乡文化服务体系进一步完善，基层文化设施实现全覆盖，文化与经济、科技密切融合，文化及相关产业增加值年均增长15%。人居环境质量明显提升。单位生产总值综合能耗完成省下达的目标任务，城镇生活污水处理率70%以上、生活垃圾无害化处理率80%以上；森林覆盖率28.5%；城区人均公园绿地面积13平方米以上。成功创建国家生态园林城市、国家卫生城市、国家环保模范城市和广东省林业生态市。民生保障水平明显提升。基本实现充分就业和人人享有社会保障。城镇居民人均可支配收入和农民人均纯收入年均分别增长10%；城镇登记失业率控制在3.5%以内；新型农村社会养老保险实现全覆盖；城镇职工和城乡居民医疗保险参保率99%以上；九年义务教育巩固率94%，高中阶段教育毛入学率90%。

　　五年看头年，开局是关键。2011年是实施“十二五”规划的开局之年，做好今年的经济社会发展工作，具有十分重要的意义。

　　2011年我市经济社会发展的总体要求是：认真贯彻党的十七届五中全会和省委十届八次全会精神，紧抓科学发展主题，围绕“转变发展方式、建设幸福湛江”这个核心，深入实施“工业立市、港口兴市、生态建市”发展战略，注重构建现代产业体系，注重基础设施建设，注重统筹城乡协调发展，注重增强自主创新能力，注重推进改革开放，注重保障改善民生，确保“十二五”发展开好局起好步，为争当粤西地区振兴发展龙头奠定坚实基础。

　　2011年我市经济社会发展的预期目标是：生产总值增长13%，人均生产总值增长11.5%，万元生产总值能耗下降3.66%；固定资产投资增长20%；港口吞吐量1.46亿吨；社会消费品零售总额增长18%；消费价格涨幅控制在4%左右；外贸进出口增长10%，实际吸收外资增长15%；地方财政一般预算收入增长15%，人均财政一般预算收入增长13.5%；城镇居民人均可支配收入和农村居民人均纯收入分别增长10%；城镇登记失业率控制在3.5%以内；城镇生活污水处理率68%、生活垃圾无害化处理率35%；人口自然增长率控制在8.03‰以内。

　　为实现上述任务目标，今年必须着力做好以下工作：

　　一、着力调整优化产业结构，进一步推进转型发展

　　以大型龙头项目为引领提升发展工业。全面启动广钢环保迁建湛江和中科炼化一体化项目建设，申报设立东海岛30平方公里石化产业园，建成晨鸣林浆纸一体化和奥里油油改煤发电项目，实施东兴石化系列产品升级项目，支持中海油扩大产能，动工建设广东冠豪特种纸及包装纸生产基地等项目。加快广州（湛江）产业转移工业园和农垦湖光工业园区、奋勇华侨工业区建设，打造工业新增长点。以“强工富县”为突破口壮大县域经济，推动重化产业链项目和承接产业转移项目向县域布局，形成若干支柱产业集群；推进佛山顺德（廉江）、深圳龙岗（吴川）省级产业转移工业园和廉江家具产业基地建设，动工或投产一批项目；支持县域国有、集体企业改制，增强县域工业发展活力。

　　以物流商务中心和特色旅游为引领提升发展现代服务业。组建和引进大型航运集团投资扩大集装箱运输及中转业务；启动建设湛江商贸物流城、东海岛综合物流园区和综合保税区，加快打造区域性航运中心和物流中心。创建农村综合产权交易中心和农海产品期货交易中心。突出推进人民大道南、海田片区商业圈和旧大天然片区至开发区中央商务区建设。促进建设广东（湛江）统筹城乡发展金融改革创新综合试验区，引进更多国内外金融机构设立分支机构或地区总部，打造金融服务区；支持市商业银行跨区域发展和有条件的农村信用社逐步改制为农村商业银行或合作银行。制定《旅游产业发展规划》，出台鼓励旅游投资优惠政策；加快霞山渔人码头、徐闻大汉三墩旅游区等27个重点旅游项目建设；支持徐闻县建设省旅游综合改革试点县、湖光岩创建国家5A景区、东海岛旅游区创建国家4A景区和特呈岛创建国家级滨海生态旅游区；评定一批星级农（渔）家乐旅游点；精心举办旅游主题活动，深化区域旅游合作，着力打造广东“黄金海岸、生态海岛、休闲胜地”旅游品牌。

　　以优势农产品基地为引领提升发展现代农业。实施《湛江市现代农业发展规划》，优化农业区域布局，着力建设绿色食品、有机食品、名牌产品、无公害农产品等优势农产品基地。加快正大（湛江）农业综合开发基地项目，打造现代农业示范样板。推进南国花卉科技园、湛江农垦国家现代农业示范区等5大现代农业项目。落实粮食安全和“菜篮子”政府责任制，稳定粮食种植面积，加强区域性湛江冬季蔬菜生产基地和廉江、遂溪两个国家生猪调出大县建设。发展深水网箱养殖产业园和远洋外海渔业基地，筹建珍珠交易中心和国家、省级珍珠良种场。培育壮大农业龙头企业、农场、专业合作社、专业大户等现代农业经营主体，推进农村土地承包经营权流转和农业机械化，提高农业产业化水平。造林20万亩，建设浆纸原料林基地300万亩。加强现代标准农田建设，完成城乡水利防灾减灾和省专项实施的病险水库除险加固工程；加快雷州青年运河灌区续建配套与节水改造工程；建成硇洲、乌石国家中心渔港，推进龙头沙、草潭、海安等国家一级渔港建设。

　　以临港产业带为引领提升发展海洋经济。完善海洋经济发展总体规划，优化布局，大力推进海洋工业、海洋运输、海水养殖、海洋捕捞、海产品加工、滨海旅游“六路并进”综合开发，形成“蓝色产业”集聚带，构建现代海洋产业体系。加速海洋科研成果推广应用。争取重大填海造地项目和涉海工程纳入省海洋功能区划统筹开发。加强海岸、海域科学利用和管理，完善海岛基础设施配套，推进罗斗沙等无人海岛保护开发。支持湛江经济技术开发区申报创建省级海洋综合开发示范园区。

　　二、着力扩大内需，进一步推进投资消费协调发展

　　加快重点项目建设。今年安排重点建设项目67项，计划投资225.71亿元，带动固定资产投资增长20%以上。建成东海岛疏港公路、南三大桥、霞山海滨码头改造和茂湛高速公路官渡立交工程；加快湛江港宝满集装箱码头、30万吨级散货码头、东海岛通用泊位一期工程、茂湛铁路、海湾大桥西连接线、鉴江供水枢纽工程以及高速公路与县域、乡镇、园区连接线建设；推进湛江港40万吨级航道、东头山岛油品码头一期、钢铁基地和中科炼化一体化项目配套码头、徐闻南山港、东海岛铁路、官渡至南三大桥一级公路、东海岛至雷州跨海大桥和南三岛至东海岛海底遂道等项目前期工作；积极支持配合建设广东西部沿海高速铁路、合浦至湛江铁路、粤海铁路湛江至海安段扩能改造项目，启动湛江国际机场迁建工程。

　　推动消费升级。认真落实中央、省扩大内需的战略方针和一系列政策措施，继续培育汽车、文化、体育、信息服务、旅游等消费热点，扩大“中国海鲜美食之都”等品牌效应，提升发展饮食服务业，优化消费结构。加强宏观调控，抑制房地产价格过快上涨，促进房地产市场健康发展。继续开展家电、汽车、农机、建材下乡和家电以旧换新活动，扩大农村消费。积极发挥各级消费者委员会作用，维护消费者权益，增强居民消费信心。

　　完善市场体系。健全资本、产权、土地、人力资源等要素市场，规划建设小家电产品及配件批发市场，继续做大汽车销售市场；在主产地建设一批大型化、高标准农副产品批发市场；建设湛江粮油物流中心和5万吨粮库。推进农贸市场升级改造，完成霞山水产品批发市场迁建，建成国联南方水产品交易中心二期工程，打造区域性海产品集散中心。

　　三、着力推动体制改革和科技人才强市，进一步推进创新发展

　　深化经济体制改革。理顺市与区权责利关系，调动区级发展经济积极性。组织实施富县强镇事权改革，增强县镇发展活力。完善财税和投融资体制。加大国有企业资源整合力度，健全国有资本经营预算制度和法人治理结构；推动市属国有企业与中央和省属企业合资合作，将国有资产经营公司建成国有资产进退平台，支持优势骨干企业上市，促进国有企业做强做大。完成华侨管理区体制改革。

　　致力发展民营经济。完善鼓励和引导民间投资政策，支持民营资本进入基础产业、基础设施、市政公用事业、社会事业、文化产业等领域；支持民营企业承接珠三角产业转移，积极参与钢铁、石化项目配套产业链建设；支持民营企业参与国有企业改革。设立市财政中小企业发展专项资金，发展融资性担保行业，推进村镇银行和小额贷款公司试点，努力解决中小企业融资难。引导民营企业加快制度、技术和管理创新，不断壮大规模、提升层次、增强实力。

　　构建科技创新平台。推动产学研合作、企业技术创新、科技成果转化、科技公共资源共享、科技公共服务5大平台建设，构筑具有集聚、示范和辐射作用的创新创意创业载体。加快湛江高新技术开发区发展，支持申报国家级高新区。规划建设赤坎科技产业园区。积极与高等院校、科研机构合作，共建技术研发和中试服务基地、共享实验室，推进科技研发孵化工程。促进专业镇技术创新与转型升级。坚持名牌带动，强化企业创新主体地位，加强品牌和管理创新，打造一批拥有自主品牌与技术的旗舰型企业和名牌产品。

　　加快发展新兴产业。依托优势产业，突破核心技术，加速培育引领和支撑未来发展的先导性、支柱性产业。大力发展新能源、新材料、节能环保、生物医药、电子信息、先进装备制造等产业。推进广东中能30万吨木薯燃料乙醇项目一期、广东粤电湛江生物质发电厂、特呈岛太阳能光伏、徐闻生物质热电联供和徐闻勇士、角尾及坡头南三、雷州东里风能发电等一批新兴产业项目，促进新兴产业规模化、集群化。

　　大力引进人才促创新。全面实施人才强市战略，以高层次、高技能产业人才为重点，统筹推进各类人才队伍建设。实施一批重大人才工程和政策，创造优良环境和条件，紧紧围绕主导产业和战略性新兴产业，突出引进一批带技术、带项目、带资金的创业创新领军人才。建立引进高层次人才基金，对有突出贡献人才给予重奖。

　　四、着力加快城镇化进程，进一步推进统筹发展

　　强化城市规划统领作用。加快编制《湛江市城市总体规划（2010-2020）》、《湛江市城市近期建设规划（2011-2015）》、《湛江市城乡发展战略规划》、《“一湾两岸”城市景观规划》、《市区城中村规划》和修编城市分区规划及控制性详细规划，协助编制《粤西城镇群协调发展规划》，启动编制《市区地下空间利用与控制规划》和《海岛开发建设规划》，建立完善的城市规划控制体系。加快县（市）城区及建制镇总体规划修编，提高城镇规划区控制性详细规划覆盖率。

　　完善城市基础设施。完成疏港大道二期、奋勇大道、滨湖路二期等7条道路工程，加快新湖大道建设以及市区3条高等级沥青道路和8条小街小巷改造，积极推动建设椹塘路、银苑路，抓好湛江大道前期工作，建设市区客运枢纽站场和公共自行车服务系统。加强市区绿道网建设，完成滨湖公园一期工程，启动银帆公园、麻章公园、体育公园、坡头区南油公园、东海岛公园和园林垃圾生态处理系统等项目，加快三岭山森林公园生态恢复整治；改建部分公园绿化，开展赤坎水库公园前期工作，年内市区新增绿地面积100万平方米以上。改造市区8条道路路灯，逐步推进城市亮化工程。

　　推动新城区开发建设。加速城市东扩，以第十四届省运会场馆建设和海湾大桥实行年票制为契机，实施海东新区首期综合开发，高起点高标准谋划一批教育、居住、商贸、金融、文化和公共服务设施项目，启动东海岸观海长廊、游艇俱乐部等项目，逐步建成“一湾两岸”城市副中心。全面落实地方与农垦合作发展框架协议，以教育产业园为切入点，推进湖光片新城区建设。加快东海岛以服务钢铁、石化基地为重点的城市化进程，打造宜工宜商宜业新城区。

　　强力推进“三旧”改造。加快海滨大道改扩建和旧大天然片、霞海港片以及霞山汉口路、东堤路、洪屋路、赤坎南华广场片旧城区集中连片改造，打造城市精品片区；加强历史文化建筑保护，推进赤坎“三民路”和霞山欧陆风情街建设，打造历史文化街区；抓住产业转型升级契机，推动城市中心区工业企业异地迁建，促进城区改造“腾笼换鸟”，打造新的城市功能区；抓好文保村、龙潮村、霞海村等城中村、城郊村改造，打造旧村庄改造示范品牌。

　　建设城管长效机制。加快城市管理体制由“条块结合”向“属地为主”管理模式转变，明确管理责任主体和执法主体，推动执法重心下移，赋予街道、社区更多的管理权限和责任，形成纵管到底、横管到边、全时制监管、全方位覆盖的管理和责任网络。完善数字化城市管理系统，构建快速反应的城市综合管理平台。加大城市建设执法督察力度，坚决依法拆除和遏制违法建筑、违规广告设施。坚持城市环境卫生治理常态化、制度化，维护良好的市容面貌。依法推进市区交通秩序整治专项行动。

　　提升城镇化水平。完善县域城镇布局，坚持城镇建设与农场场部、工业园区、商住小区建设有机结合，推动人口和产业向“一城数镇”集聚。全面落实扩大中心镇管理权限，努力把中心镇培育成为县域人口集中、产业集聚、功能集成的新载体。放宽城镇户籍限制，建立城乡统一的户口登记制度，落实农民工进城入户积分制，促进人口到城镇落户。以推进农业产业集中发展、农民集中居住、土地集中经营为重点，加快建设农村新社区，进一步提高新农村建设水平；切实抓好农村劳动力技能培训和转移就业,把符合条件的农业转移人口逐步转为城镇居民。

　　五、着力扩大开放，进一步推进外向发展

　　转变外贸发展方式。实施科技兴贸战略，鼓励出口企业加强技术改造、自主研发和技术引进，申报国家、省级品牌，培育以技术、品牌、营销网络为核心竞争力的新优势。提升加工贸易产业层次，扩大服务贸易，促进出口结构转型升级。推动出口市场多元化，优化出口市场结构。进一步完善口岸基础设施。支持开发区申报海关特殊监管区，抓好湛江保税物流中心和保税港区审批跟踪工作。

　　提高招商引资实效。推动招商引资向招商选资转变，着力引进一批投资总量大、科技含量高、带动能力强、财税贡献多、能耗污染低的大项目。强化产业链招商和工业园区招商，精心筛选推出一批以钢铁、石化等主导产业为重点的招商项目，着力引进世界500强企业和国内战略投资者，推动产业升级。加强招商引资载体建设，重点完善开发区、工业园区基础设施和配套条件，保障项目用地，提高招商实效。积极引导湛商回归。鼓励和支持外商投资企业增资扩产。

　　推进区域对接合作。加强与珠三角合作，深入推进“双转移”。拓展与广西北部湾经济区合作领域，主动融入海南国际旅游岛开发建设，推进与西南各地共建出海大通道。密切粤西各市合作，合力培育“湛茂阳经济发展圈”。落实粤港澳合作和两岸经济合作框架协议，强化与港澳互利合作，加快湛江海峡两岸农业合作试验区建设。积极开拓国际友城。推进我市与中国—东盟商务理事会战略合作，举办中国—东盟中小企业合作论坛。

　　六、着力加强生态建设，进一步推进绿色发展

　　节约利用资源能源。完善资源综合利用鼓励政策，加快工业、建筑、交通、公共机构节能降耗技术改造；实施差别性价格促进生产结构转变，抑制高耗能产业过快增长，提高能源利用效率。健全工业及建设用地招拍挂出让制度，探索城乡建设用地增减挂钩和农村集体经营性建设用地依法公开转让机制，严格耕地保护，促进节约集约用地。支持重点领域节水工程建设，开征垃圾处理费和水资源费，稳步推进居民阶梯式水价和阶梯式电价，促进节水节电。加强稀土等资源保护和科学合理开发利用。

　　保护生态环境。进一步加强对主要江河、重要水源涵养区及饮用水源、战略性水源保护；推进南柳河和北桥河治理。强化市区海域海水养殖污染治理，定期发布海洋环境质量公报。积极创建国家级生态示范区、广东省林业生态市（县）、各级生态示范村镇和绿色社区，加快建设沿海防护林、交通绿色长廊、江河水源涵养林等七大生态带和区域绿道，构建沿海绿色生态屏障；继续选择200条自然村开展林业生态文明万村绿大行动。抓好农村村场改造，推进新民居建设。努力完成国家确定的主要污染物减排任务，重点抓好国控、省控污染源环境监管，推进环保收费改革，实现达标排放。建成坡头区、东海岛污水处理厂，加快建设赤坎污水处理厂三期、开发区平乐污水处理厂及中心镇污水处理厂工程；筹划建设市区冯村垃圾处理场三期和垃圾焚烧发电厂及县（市）城区、中心镇垃圾无害化处理场。扎实推进创建国家环保模范城市和国家卫生城市，争取年内取得全面进展。

　　发展循环经济。以提高资源产出效率为目标，实行生产者责任延伸制度，推进生产、流通、消费各环节循环经济发展。发展生态循环农业，建设一批立体种植养殖、轮作复种等示范项目和示范区。发展循环工业，鼓励企业间通过共享资源、废弃物利用等途径发展循环经济，推进清洁生产，创建循环经济示范企业；重点发展钢铁、炼化一体化循环经济产业，创建东海岛国家级循环经济示范区。建成林浆纸一体化生产基地。强化资源综合利用和再生利用，建设粤西（吴川）再生资源产业基地。

　　七、着力保障和改善民生，进一步推进和谐发展

　　促进教育公平发展。制定实施《湛江中长期教育改革和发展规划纲要(2011—2020)》、《湛江市学前教育三年行动计划》和《湛江市城区教育专项规划》。完善学前教育体制机制，多途径整合学前教育资源，着力解决幼儿“入园难”问题。开展以集聚办学为主要内容的新一轮布局调整，加快建设义务教育规范化学校，全面完成校舍安全建设工程，推进市四中新校区建设，动工建设首批进入市职业教育基地学校。巩固提高普及高中阶段教育成果，高中阶段教育毛入学率达到87%以上。加强初中学生“防辍”工作，完善扶贫助学机制，逐步解决外来务工人员子女入学问题。支持发展民办教育。

　　建设文化强市。加强社会主义核心价值体系教育，大力弘扬“开放兼容，勇立潮头”的湛江人精神。实施基层文化设施全覆盖工程，加快完善市、县、乡镇、村四级公共文化设施网络。逐步建设湛江粤剧院、歌剧院、文化艺术中心、中心文化广场、文化主题公园、新闻大厦等市区标志性文化项目和文化生态保护区，推进国家重点文化惠民工程项目，增强公共文化产品和服务供给能力。推动具有传统优势的文化产业培育自主品牌，延伸产业链条，发展一批文化产业集团。启动建设文化创意产业基地。

　　发展体育事业。加快建设第十四届省运会体育场馆、配套设施和水上运动中心，加强竞技体育后备人才培养，创办湛江跳水运动学校，组团参加全国第七届城市运动会，举办第五届湛江海上国际龙舟邀请赛，办好市第十二届体育节。

　　优化医疗卫生服务。加快建设城乡医疗卫生服务体系，巩固和完善城乡居民基本医疗保险一体化制度。推进医药价格改革和单病种收费改革试点，政府办基层医疗机构全面实行国家基本药物零差率销售。围绕公益性核心目标推进公立医院改革发展，支持湛江中心人民医院迁建和广东医学院附属医院、市第一中医院、市妇幼保健院扩建，筹建市医疗紧急救援指挥中心、湛江长者康复医院和市口腔专科医院。继续实施卫生信息化工程，提高城乡居民健康档案建档率。

　　扩大城乡就业创业。全面落实积极的就业政策，综合运用财政、产业、社会和公共服务等就业援助政策，建设惠及全民的就业体系。以钢铁、石化和林浆纸一体化基地为平台，鼓励发展优势劳动密集型产业，扩大就业规模，改善就业结构。大力开发社区就业岗位和公益性岗位，建设高校毕业生创业基地，抓好失业人员再就业培训和农村劳动力转移培训。推进城乡就业援助工程，完善失业保险与免费技能培训、促进就业联动机制以及集保障生活、预防失业、促进就业三位一体的失业保险制度；积极做好“零就业家庭”就业帮扶工作。

　　提高社会保障水平。健全城乡一体化社会保障体系，完善企业职工养老保险市级统筹，进一步抓好社会保险扩面征缴工作，推进地税全责征收社保费。年内企业养老保险、失业保险、医疗保险（含城乡居民医保）、工伤保险、生育保险参保人数分别达67万人、34万人、650万人、29万人、34万人。抓好企业职工基本养老保险关系转移接续和事业单位养老保险改革，建立80周岁以上高龄老人津贴和重特大疾病救助制度。推进坡头区省新型农村养老保险制度试点，年内中老年农村居民参保率达40%以上。推行被征地农民社会保障制度，逐步提高城镇与农村最低生活保障标准。继续实施助残工程。提高住房公积金使用率，增强职工购建住房能力，加快城镇保障性住房特别是廉租房、公租房建设，不断满足群众多层次住房需求。

　　抓好扶贫“双到”工作。坚持扶贫“双到”工作与建立现代产业体系、双转移、新农村建设、创先争优活动、农村弱势群体救助救济相结合，采取输血与造血、扶贫与扶智、思想扶持与物质扶持、短效与长效相结合的方式，提高帮扶实效，年内实现贫困村贫困人口80%的稳定脱贫目标。扩大贫困家庭青壮年劳动力就业与创业培训和子女免费技术教育，实现培训一人、就业一人、脱贫一户。广泛发动社会各界开展“广东扶贫济困日”活动。

　　协调发展各项事业。加大力度做好人口和计划生育工作，稳定低生育水平，促进人口长期均衡发展。强化税收征管，提高征管效能。加强国防后备力量特别是基层民兵预备役建设，全力争创“双拥模范城”全国四连冠、全省五连冠。抓好人防信息化、组织指挥体系和人防工程建设。推进市档案馆建设，加快档案管理数字化和信息化。完成第二轮新地方志修编。切实抓好统计、外事、侨务、民族宗教、对台、地震、老区、粮食、供销社、气象、社会科学和妇女儿童、老龄、关工委以及村级换届选举等工作。

　　推进平安湛江建设。抓好各级应急指挥平台建设，提高应对公共突发事件能力。完善社会矛盾大调解机制，建成市级综治信访维稳中心和人民群众来访接访厅，完善市、县、镇、村四级群众工作网络和县、镇、村三级综治信访维稳平台，推动规模以上企业建设综治信访维稳工作站，及时化解社会矛盾纠纷与不稳定因素。加大食品、药品、保健食品、医疗器械等监管力度，确保人民群众饮食和用药安全。落实低收入群众价格补贴及农业各项价格扶持政策，加强物价调控，稳定市场供应和价格秩序。落实党政干部安全生产“一岗双责”和企业安全生产主体责任，强化重点行业、重点领域安全生产标准化建设，努力实现省政府下达的安全生产控制目标。建设反走私和海防监控指挥中心。加强社会治安综合治理，加快社会治安视频监控网络建设，推进群防群治工作；依法严厉打击重大刑事犯罪尤其是涉黑恶、涉枪、涉毒和“两抢一盗”等严重违法犯罪活动，增强群众的安全感。

　　办好20件民生实事。城镇新增就业6.5万人，城镇失业人员实现再就业4万人；完成农村劳动力免费技能培训4万人，新增农村劳动力转移就业13万人；培训旅游从业人员1万人、组织旅游企业为农村富余劳动力提供就业岗位2000个；为市区失业下岗人员开辟6个限时性市场；在市区兴办2个平价农贸市场和一批平价商店；完成15个乡镇中小学布局调整项目，建成129幢、20万平方米中小学校舍安全工程，力争70%以上外来工子女入读公办学校；完成和启动20家乡镇卫生院改扩建；新增LNG小型公交车200台、出租车300台投入运营；完成农村公路硬底化改造850公里，新建农村客运站20个、候车亭300个，具备条件的行政村全部通客运班车；改造南三岛等5个陆岛交通码头和8个乡镇横水渡码头；建成廉租房430套2.15万平方米、公共租赁住房250套1.38万平方米；完成2000户历史遗留房地产权登记；建设水库移民新村85条，解决4000户、1.8万水库移民住房困难；改造农村贫困群众危房12580户；实施农村饮水安全工程15宗，解决43.14万人饮水问题；建成农家书屋300个；实现每条行政村每月放映一场公益电影；新建和改扩建乡镇敬老院（中心）20所；农村五保供养标准提高到150元以上，城乡低保标准分别提高到200元和125元以上；创建40个防灾减灾示范社区。

　　八、着力提高行政执行力，进一步推进率先发展

　　更加注重依法行政。全面贯彻《法治湛江五年规划》，推进依法治市，把依法行政贯穿到政府工作的各个领域、各个环节，严格按照法定程序、法定权限、法定职责行使人民赋予的权力。广泛开展“六五”普法教育，提升全民法律意识。抓好政府部门依法行政能力建设，加强行政执法指导和监督，严格按程序和规则办事。加强规范性文件备案审查。健全公众参与、专家论证和政府决策相结合的行政决策机制。深化政务公开，强化政务服务建设，努力把各级政府网站建成政府信息公开第一平台。认真落实人大及其常委会各项决议决定，坚持向人大及其常委会报告工作，自觉接受监督；主动加强同人民政协的联系，支持政协委员发挥参政议政作用；积极办理人大代表议案、建议和政协委员提案。

　　更加注重提高效能。全面实施《湛江市市直机关科级以下公务员年度考核实施细则》，深化机关效能和作风建设。推进第四轮行政审批制度改革，进一步精简审批项目，减少环节，缩短时限；完善以行政服务中心为主要载体的政务服务体系，加快启动市行政中心建设，推行部门行政审批和服务全面进驻窗口，推广业务集中受理和并联审批，把行政服务中心建成集行政审批、政务公开、便民服务、效能监察为一体的政务服务综合平台。继续完善行政审批和重点工作、重点项目督查督办电子监察系统，实现全程监督，提高行政办事效率。

　　更加注重服务创新。在加强和改善经济调节、市场监管的同时，更加注重社会管理和公共服务，确保政府管理服务到位。深入开展“创业服务年活动”，重点在项目引进、立项、报批、跟踪上搞好服务，在为创业者提供创业培训、信息咨询、融资贷款、人才引进、政策扶持上搞好服务，在营造政府鼓励创业、社会支持创业、劳动者积极创业的浓厚氛围上搞好服务，努力打造对内具有凝聚力、对外具有吸引力的投资创业环境。

　　更加注重廉政建设。按照党风廉政建设责任制的要求，切实抓好政府系统党风廉政建设和反腐败各项工作。坚持开展纪律教育月活动，建立健全预防腐败的各项制度，在工程招标、土地出让、产权交易、政府采购、征地拆迁、“三旧”改造以及社保基金、住房公积金和政府专项资金管理等重点领域加强督查。严格规范公共财政支出，深入开展公车治理，继续压减因公出国（境）、公务接待、公务购车用车经费。推进领导干部经济责任审计，加强政府投资项目跟踪审计。坚决纠正损害群众利益的不正之风，严肃查处违法违纪案件。各级政府机关和公务员要坚持以人为本、行政为民，牢固树立群众观点，全心全意为人民服务；自觉遵守廉洁自律各项规定，做到以廉生明、以公树威、以苦励志、以俭修身、以慎施政，用实际行动当廉政建设的表率，做人民群众满意的公仆。

　　各位代表，湛江未来五年的发展蓝图已经绘就，一个新的飞跃正展现在我们面前。加快转变发展方式、建设幸福湛江，是时代赋予我们的光荣使命，目标鼓舞人心，任务催人奋进。让我们在中共湛江市委的正确领导下，坚定信心、振奋精神，锐意进取、开拓创新，扎实工作、苦干兴市，为争当粤西地区振兴发展龙头、开创更加美好幸福的未来而努力奋斗！
